# The Role of Thiazolidinediones in the Amelioration of Nonalcoholic Fatty Liver Disease: A Systematic Review

**DOI:** 10.7759/cureus.25380

**Published:** 2022-05-27

**Authors:** Andrew Ndakotsu, Govinathan Vivekanandan

**Affiliations:** 1 Internal Medicine, California Institute of Behavioral Neurosciences and Psychology, Fairfield, USA

**Keywords:** non alcoholic fatty liver, non alcoholic steatohepatitis, nash, nafld, pioglitazone, thiazolidinediones

## Abstract

Non-alcoholic fatty liver disease (NAFLD) is a broad term encompassing hepatic steatosis and non-alcoholic steatohepatitis (NASH), a form of chronic hepatitis. This may, unfortunately, lead to terminal complications like cirrhosis and hepatocellular carcinoma (HCC). NAFLD is strongly associated with obesity, type 2 diabetes (T2DM), hypertension, and metabolic syndrome. The growing prevalence of NAFLD, its associated conditions, and its complications are alarming. The insulin sensitizer group "thiazolidinediones" has shown some therapeutic benefits in this condition. This systematic review is intended to focus on the clinical efficacy of this group in patients with NAFLD, employing PubMed, Google Scholar, and the Cochrane Library as databases.

We discovered 10 randomized control trials (RCTs; nine involving pioglitazone and one involving rosiglitazone) involving 887 participants. All studies varied in duration from 6 to 24 months. Most of the involved trials had a small number of participants, and the intrinsic quality of the studies was mixed. Pioglitazone consistently improved histological parameters and normalized liver transaminases, although evidence supporting the benefits of other drugs in this class was minimal. Thiazolidinediones, particularly pioglitazone, have proven efficacious in patients with NAFLD/NASH. However, more extensive trials need to be carried out to investigate this drug class's benefits further. Unfortunately, this drug has attendant side effects like weight gain and fractures, limiting its widespread use; hence, careful selection for likely candidates is imperative.

## Introduction and background

The amassing of excessive fat within the liver, which may lead to consequent hepatic injury after alcohol consumption has been excluded, is termed non-alcoholic fatty liver disease (NAFLD). Non-alcoholic fatty liver disease encompasses non-alcoholic fatty liver (NAFL), non-alcoholic steatohepatitis (NASH), and NASH cirrhosis [1]. One-fourth of the world's populace is said to have NAFLD [2]. In addition, this disease entity has the natural capacity to advance to cirrhosis, hepatocellular carcinoma (HCC), and death [3]. Over the last 20 years, the recent surge in NAFLD has seen this condition reveal itself as one of the foremost contributors to chronic liver disease; this unfortunate increment has challenged the significant strides made in managing hepatitis B and C [3]. Due to the intimate relationship that exists between NAFLD, type 2 diabetes mellitus (T2DM), obesity, and attendant insulin resistance, it is no surprise that NAFLD has continuously expanded in prevalence, making it the most common cause of chronic liver disease, of which 25% progress to NASH. Hence, in the not-so-distant future, NAFLD may very well be one of the primary indications for a liver transplant [4]. Many studies have appreciated that NAFLD occurs more frequently in patients that do have T2DM and, in addition, manifests at a greater severity. Furthermore, this cohort of the diseased population has a larger propensity to progress to cirrhosis and hepatocellular carcinoma besides the established cardiovascular morbidity attached to diabetes mellitus [5-7]. This disclosure highlights that we may have unconsciously been helping our course as we intensify our efforts, particularly in the primary prevention of T2DM. Thus, it is of no surprise that interventions like lifestyle modifications and insulin sensitizers have, to various degrees, been tagged as beneficial among patients with NAFLD/NASH.

Obesity is also an established risk factor for NAFLD; in fact, its prevalence among the obese population has been estimated to be between 30% and 37% [8]. Obesity plays a crucial feature in the metabolic syndrome, of which its epicenter is the phenomenon called insulin resistance. Insulin resistance is intrinsic in the pathophysiology of T2DM. NAFLD interestingly is also associated with approximately an increased risk of developing type 2 diabetes mellitus; the magnitude of this risk may be directly proportional to the severity of the underlying liver disease [9]. Persistently elevated glucose levels seen in T2DM are hall-marked by glucotoxicity, and there is an expanded predominance of NASH in patients with T2DM [9]. Similarly, lipotoxicity is tightly affiliated with T2DM and hepatic dysfunction [9]. Thiazolidinediones, a pharmacological group that combats insulin resistance and its attendant challenges, thus proposes a beneficial effect in that it antagonizes and inhibits lipolysis. It is also involved in redistributing fat within the body [10]. Although we can appreciate the increase in prevalence, an accurate picture of the disease prevalence has not been best captured because of the different modalities of diagnosis (liver ultrasonography, computed tomography, and magnetic resonance imaging) and their respective sensitivities; however, the gold standard remains liver histology [4]. The Italian Association for the Study of the Liver recommended that NAFLD could be diagnosed by a patient's clinical history, physical examination, investigations (full blood count, liver function tests, metabolic panels), and abdominal ultrasound scan. They further put forward that the following patients are "high-risk" for NASH: obese patients, patients with diabetes mellitus, and patients aged >45 years and, thus, should be investigated with a liver biopsy. Additionally, they also recommended a biopsy for patients with lifestyle-resistant NASH when followed via laboratory parameters after approximately a year of observation [11]. In the US, a hepatic ultrasound is widely used, although a major limiting factor to this modality is its innate ability to miss mild fatty accumulation [12]; therefore, a more sensitive modality is magnetic resonance imaging, which offers its flexibility and accuracy; however, its cost price is also a major limiting factor. Even though the invasiveness of the gold standard test has hampered the reality of having a large-scale clinical trial with these subjects, several studies have been conducted utilizing and analyzing the effect of insulin sensitizers, especially thiazolidinediones, on patients with NAFLD/NASH.

Consequently, various studies, including systematic reviews and meta-analyses, have demonstrated the beneficial effects of thiazolidinediones on NASH and its possible attendant sequelae [13,14]. Thiazolidinediones exploit the influence that serum adiponectin has on insulin resistance. Serum adiponectin antagonizes the development of insulin resistance both in the liver and systemic tissues; it also tones down hepatic inflammation and fibrosis adiponectin. Apart from that, adiponectin has a prognostic value because it correlates inversely with the degree of steatosis and NAFLD severity [15]. Relative to healthy controls, serum adiponectin is lower in patients with NASH by greater than 50% [16]. To further our claim, serum adiponectin has been demonstrated to increase after the utilization of thiazolidinedione [17,18]. This systematic review aims to provide insight into the benefits of this class of drugs and provide credence to their use in combating one of the major players in the prevalence of chronic liver disease.

## Review

Method

The research method utilized strictly obeyed the guidelines laid down by the Preferred Reporting Items for Systematic Reviews and Meta-analyses (PRISMA) [19]. An extensive systematic search of various electronic databases was undergone to fetch relevant articles. The databases used were PubMed, Google Scholar, Virtual Health Library, and Cochrane Library. Excavation from the aforementioned databases was done via relevant keywords and Medical Subject Heading (MeSH) terms. This technique helped to generate accurate and pertinent articles about the research topic. The keywords used include thiazolidinediones, pioglitazone, rosiglitazone, troglitazone, nonalcoholic steatohepatitis, nonalcoholic fatty liver disease, nonalcoholic steatohepatitis, fatty liver disease, or liver disease.

Search Strategy: Including MeSH terms and Keywords

Thiazolidinediones OR Pioglitazone OR Rosiglitazone OR Troglitazone OR ("Thiazolidinediones/administration and dosage"[Mesh] OR "Thiazolidinediones/adverse effects"[Mesh] OR "Thiazolidinediones/agonists"[Mesh] OR "Thiazolidinediones/antagonists and inhibitors"[Mesh] OR "Thiazolidinediones/blood"[Mesh] OR "Thiazolidinediones/chemical synthesis"[Mesh] OR "Thiazolidinediones/chemistry"[Mesh] OR "Thiazolidinediones/etiology"[Mesh] OR "Thiazolidinediones/immunology"[Mesh] OR "Thiazolidinediones/metabolism"[Mesh] OR "Thiazolidinediones/pharmacokinetics"[Mesh] OR "Thiazolidinediones/pharmacology"[Mesh] OR "Thiazolidinediones/statistics and numerical data"[Mesh] OR "Thiazolidinediones/therapeutic use"[Mesh]) AND Non alchoholic steatohepatitis OR Non-alchoholic fatty liver disease OR non alchoholic Steatohepatitides OR Fatty liver disease OR Liver disease OR "Non-alcoholic Fatty Liver Disease"[Mesh].

Inclusion and Exclusion Criteria

The articles identified were challenged against our inclusion criteria, which were: participants of all age groups, all sex, and all ethnicity with NASH. Types of studies included randomized control trials (RCTs) that involved the following interventions: thiazolidinediones, rosiglitazone, and pioglitazone administered at any dose, route, or duration, given alone or as a combination (versus no intervention, placebo, or any other medication). We considered the following outcomes as adverse events: liver cirrhosis, other liver-associated dysfunctions, cardiovascular events, and new-onset diabetes mellitus. Articles involving animals were excluded.

Studies Identification

Two researchers independently evaluated the articles generated by the above search strategy; their titles and individual abstracts were screened for relevance to this review’s theme. The full texts of the remaining studies were challenged against our set inclusion criteria independently by both researchers. In areas of dispute, both researchers discussed the intrinsic characteristics of the studies, like their relevance to our eligibility criteria, study design, and outcomes measured to reach common ground. Studies that did not meet the inclusion criteria were excluded.

Data Extraction and Quality Assessment

Reviewers collected data independently using a specially designed form which included the following parameters: a brief description of the study, its respective study design, traits of selected patients, intervention employed, and outcomes based on our inclusion criteria. A discussion was arranged in case of differences between the data collected. The Cochrane risk of bias tool was employed to critically evaluate the integrity of the clinical trials. A summary of this is given in Table 1.

**Table 1:** A summary of the Cochrane risk of bias tool.

Paper trait	Sanyal et al. [20]	Belfort et al. [21]	Aithal et al. [22]	Ratziu et al. [23]	Sanyal et al. [24]	Promrat et al. [25]	Sharma et al. [26]	Rana et al. [27]	Anushirvani et al. [28]	Cusi et al. [29]
Random sequence generation (selection bias)	Low risk	Low risk	Low risk	Low risk	Low risk	Low risk	Low risk	Low risk	Low risk	Low risk
Allocation of concealment (selection bias)	Low risk	Low risk	Low risk	Low risk	Low risk	High risk	High risk	Low risk	Low risk	High risk
Blinding of both the participants and evaluators (performance bias)	Low risk	Low risk	Low risk	Low risk	Low risk	High risk	High risk	Low risk	Low risk	High risk
Blinding of assessment during outcome collection (detection bias)	Low risk	Low risk	Low risk	Low risk	Low risk	Low risk	Low risk	Low risk	Low risk	Low risk
Incomplete outcome data (attrition bias)	Low risk	Low risk	Low risk	Low risk	Low risk	Low risk	Low risk	Low risk	Low risk	Low risk
Selective reporting (reporting bias)	Low risk	Low risk	Low risk	Low risk	Low risk	Low risk	Low risk	Low risk	Low risk	Low risk
Other bias	Low risk	Low risk	Low risk	Low risk	Low risk	Low risk	Low risk	Low risk	Low risk	Low risk

Results

**Figure 1:**
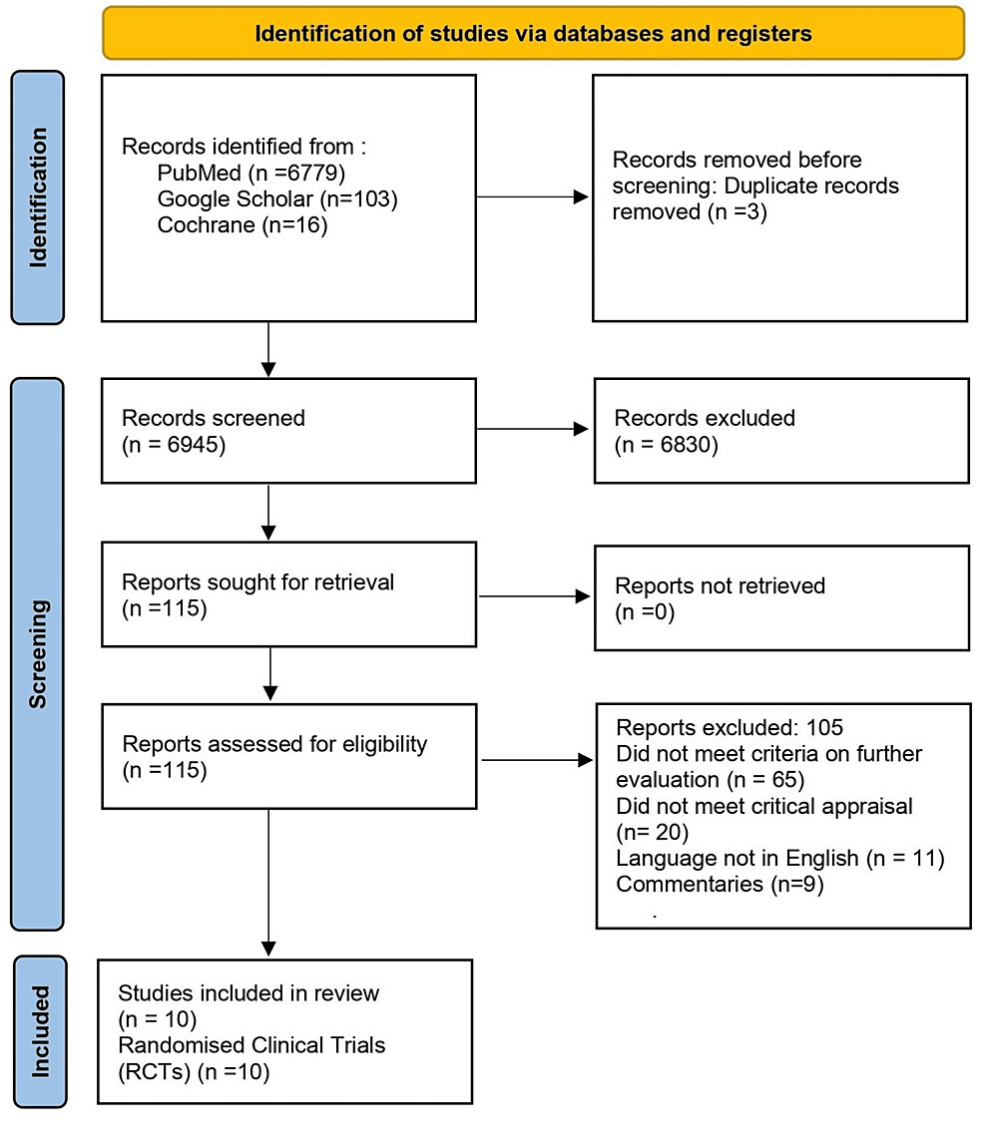
PRISMA, Preferred Reporting Items for Systematic Reviews and Meta-Analyses.

A total of 6948 studies were obtained from the various databases. Three duplicates were discovered and subsequently removed via the reference manager, Mendeley. Two separate authors screened all 6945 articles left based on their respective titles and abstracts. 4025 articles were excluded based on title screening, and then 2805 studies were excluded by the screening of their corresponding abstracts. 115 articles were then assessed for eligibility. This resulted in 105 articles being removed due to the reasons given in Figure 1. The articles left were appraised critically based on their intrinsic qualities, leaving us with 10 studies. All studies included were appraised with the aid of the Cochrane risk of bias tool.

Trials Involving Thiazolidinediones

Table 2 below depicts relevant studies that used the thiazolidinedione class medications as an intervention in patients who had nonalcoholic fatty liver disease.

**Table 2:** Finalized studies showing the dose of pioglitazone and its effect on liver cells.

Trials	Dose of drug	Duration of treatment	Participant number	Subjects with diabetes in percentage (%)	Histology	Liver enzymes	Diet counseling
Sanyal et al. [20]	Pioglitazone 30 mg	6 months	20	0	Improved	Improved	Present
Belfort et al. [21]	Pioglitazone 45 mg	6 months	55	48	Improved	Improved	Not present
Aithal et al. [22]	Pioglitazone 30 mg	12 months	74	0	Improved	Improved	Present
Ratziu et al. [23]	Rosiglitazone 4 mg for the first month 8 mg subsequently	12 months	63	31	Improved	Improved	Not present
Sanyal et al. [24]	Pioglitazone 30 mg	24 months	247	0	Improved	Improved	Not present
Promrat et al. [25]	Pioglitazone 30 mg	12 months	18	0	Improved	Improved	Present
Sharma et al. [26]	Pioglitazone 30 mg	6 months	60	0	Improved	Improved	Present
Rana et al. [27]	Pioglitazone dosage not given	6 months	98	0	not given	Improved	Present
Anushiravani et al. [28]	Pioglitazone 15 mg	3 months	150	0	Not given	Improved	Not present
Cusi et al. [29]	Pioglitazone 45 mg	18 months	101	51	Improved	Improved	Present

Discussion

The American Association for the Study of Liver Diseases (AASLD) has put forward that patients without hepatic fibrosis on histology be started primarily on lifestyle modifications and pharmacotherapy that control the progression of co-morbidities like obesity, diabetes mellitus, etc. AASLD further stated that only patients with histologically proven NASH and fibrosis should be started on pharmacotherapy [30]. However, the apparent clinical reluctance to routinely biopsy patients with multiple obvious risk factors remains. Furthermore, obese patients frequently have impaired glucose tolerance or diabetes mellitus, mainly stemming from insulin resistance [31]. As stated earlier, insulin resistance is a primary culprit in the pathophysiology of NAFLD/NASH; hence, drugs that target this pathway have shown great promise in the resolution of NASH [32]. The pioneer human study on the role of thiazolidinediones in NAFLD, which makes it worthy of mention, involved the use of thiazolidinediones against NASH, was done by Caldwell et al. in 2001; it was a pilot study. Troglitazone (since withdrawn for its causation of acute hepatocellular injury) was the intervention used for ten patients who possessed biopsy-proven NASH. However, there was a significant change in histologic comparison before and after therapy; however, results were associated with enhanced aminotransferase levels [33]. Three years later, Sanyal et al. conducted an open-labeled RCT. Their goal was to challenge the efficacy and safety of vitamin E alone against the combination of vitamin E and pioglitazone in patients with NASH. Patients with diabetes or cirrhosis were excluded from the study. Interestingly, the combination of pioglitazone and vitamin E proved superior, as evidenced by an improvement in NASH histology [20]. A modified Brunt scoring system was used to objectively distinguish the histologic changes in the liver at the end of the study duration [34]. In 2006, Belfort et al. conducted a placebo-controlled trial of pioglitazone plus a calorie-restricted diet in patients with biopsy-confirmed NASH. All subjects who participated in the study (55 subjects) were overweight (BMI greater than 25 kg/m^2^) and had either impaired glucose tolerance or type 2 diabetes based on a 75-g oral glucose tolerance test. The treatment group was administered 30 mg of pioglitazone daily for the first and second months, after which it was subsequently increased to 45 mg/day and maintained till the end of the study duration [21]. Biopsy changes in liver histology were assessed via the NAFLD Activity Score (NAS) and Fibrosis Staging [35]. Aithal et al. used pioglitazone as the only intervention in a randomized, placebo-controlled trial. In contrast to Belfort et al., the subjects of these studies were non-diabetic subjects with NASH, just like Sanyul et al. [24]. The mean BMI in the control group was 30.8 kg/m^2^ [standard deviation (SD) 4.1 kg/m^2^] in the control group, while the mean BMI was 29.8 kg/m^2^ (SD 3.0 kg/m^2^) in the treatment group. Aithal et al. made use of the NASH histological grading system of Brunt et al. [34]. On histology, markers of NASH-related liver abnormalities- steatosis, hepatocyte injury, lobular inflammation, Mallory bodies, and fibrosis were all improved after a 12-month period of 30mg/day of pioglitazone. A multi-center double-blind placebo-controlled randomized controlled trial was carried out in 2010 by Sanyal et al.; the goal of the study was to evaluate the difference between vitamin E and pioglitazone with placebo in NASH. This study had the highest number of participants relative to other earlier studies, and unlike other studies, participants were stratified into three groups based on their respective body mass index (BMI). The mean BMI in all three groups ranged between 34 and 35 kg/m2. Interestingly, remarkable improvements in histological matrices were seen in both vitamin E and pioglitazone arms; however, between both non-placebo interventions, there was no significant difference in each of the histological parameters [24]. An open-labeled randomized control trial carried out by Sharma et al. in 2012 compared the efficacy of pioglitazone, pentoxifylline, and pioglitazone on hepatic biopsy and metabolic factors of patients with NASH [26]. Liver histology was assessed based on the method of Brunts et al. for assessing necrosis and inflammatory grade and also the stage of fibrosis [34]. In the pioglitazone arm, there was a significant improvement in hepatic steatosis [26]. This finding was noticed by Promrat et al. (p < 0.001) [25]. Ratziu’s study in 2008 demonstrated a significant reduction in hepatic steatosis between both groups (47% vs. 16%; p=0.014) using Rosiglitazone as an intervention. This marked reduction in steatosis was accompanied by stabilizing transaminase levels (38% vs. 7%; P=0.005) [23].

Liver Histology

When working up a patient with the possibility of having NASH, it is imperative that all other causes of hepatopathy are excluded. The AASLD conference in 2002 put forward a group of histological features that they found imperative and, hence, recommended them in the diagnosis of NASH [36]. These histopathological features are summarized in Table 3 [36].

**Table 3:** Histological abnormalities in non-alcoholic steatohepatitis. [36]

Essential components	Features
Steatosis	Macrosteatosis greater than microsteatosis in zone 3 of the hepatocytes.
Lobular inflammation	Mixed and mild inflammation; marked by the presence of both mononuclear and polymorphonuclear white blood cells.
Ballooning of hepatocytes	Most apparent near steatotic liver cells; typically located in zone 3 of the hepatocytes. It is usually present but not necessary for making a diagnosis.
Fibrosis	Peri-sinusoidal fibrosis in zone 3 of hepatocytes.
Glycogenated nuclei	Hepatocellular glycogenated nuclei in zone 1.
Lipogranulomas	Presence of lipogranulomas in the hepatic lobules; which are usually small.
Periodic acid shiff stain	Presence of Kupffer cells stained by periodic acid -Schiff. Presence of periodic acid stained alpha-1 antitrypsin globules in the periportal hepatocytes.
Fat cysts	May be present but not necessary for making a diagnosis.
Mallory-Denk bodies	Observed in ballooned hepatocytes - majorly in zone 3 of the hepatocytes; however, may be seen in zone 1 in diabetes and during amiodarone use. Typically poorly appreciated, may require special immunostaining for enhancement.
Iron deposition	Seen usually in grade 1, best detected by Prussian blue stain
Hepatocyte organelle changes	Large intra-cytoplasmic mitochondria
Cholestasis	Acute cholestasis - presence of biliary plugs. Chronic cholestasis - ranges from the presence of marked ductal lesions, ductal proliferation, accumulation of copper granules in periportal hepatocytes, and biliary duct loss.

Despite these vast arrays of possible histologic features associated with NASH, four stand out: steatosis, hepatocellular ballooning, lobular inflammation, and fibrosis of the peri-sinusoidal areas [35]. These popular features are pretty similar to the grading and staging system proposed in 1999 by Brunt et al. [34]. However, the presence of fibrosis is not useful in the diagnosis of NASH despite its usual presence [35]. The NASH Clinical research network made some modifications to the classification of Brunt et al. and came up with the NAFLD activity score; this score proves helpful, mainly because it can be used for the full spectrum of NAFLD [35].

Effect of Thiazolidinedione’s on Liver Histology in NASH

Pioglitazone has shown remarkable improvement histologically in patients with NASH. This was demonstrated by Aithal et al. [22]. In addition, hepatocellular ballooning, lobular inflammation, and peri-sinusoidal fibrosis were also improved in histology. Vitamin E was postulated to have similar effects on the histological picture in NASH patients. Sanyal et al. compared these proposed effects with pioglitazone and found that pioglitazone was inferior to vitamin E when compared to each other in respect to hepatocyte ballooning, but lobular inflammation was better resolved in patients who received pioglitazone [24]. In addition, there was a much greater statistically significant benefit in steatohepatitis resolution in the arm that received pioglitazone versus the arm that received vitamin E [24]. A significant limitation of this study was the use of a known anti-diabetic drug among participants who were established as non-diabetic. Another noteworthy limitation is that some of the participants may not have had true NASH, thus grossly under-powering the study. However, the randomized control trial of Sanyal et al. involving pioglitazone in NASH patients pointed out that pioglitazone does have a significant effect on hepatocyte ballooning necrosis (p=0.02) [24]. Sharma et al. support the findings of Sanyal et al. on the positive impact of pioglitazone on lobular inflammation and a statistically significant improvement in Brunt's grade (p=0.005) [26]. Furthermore, Belfort et al., Sanyal et al., and Sharma et al. found zero improvements in fibrosis [21,24,26]. For patients with pre-diabetes and diabetes, Cusi et al. noticed an improvement in hepatic histology among patients who took pioglitazone [29]. Among patients without DM, Ratviu et al. examined the long-term effect of rosiglitazone in the Fatty Liver Improvement with Rosiglitazone Therapy (FLIRT) 2 trial and reported that longer therapy does confer no additional benefit upon the regulation of insulin sensitivity and transaminase levels, highlighting that other pathways causing this disease need to be explored and exploited [23]. Both Rana et al. and Anushiravani et al. carried out their respective studies on non-diabetics. Pioglitazone was the insulin sensitizer intervention; in these two studies, histological biopsies were not taken [27,28].

Liver Function Tests

In all trials involving pioglitazone as an intervention, there was a significant reduction in ALT levels, except in the first study by Sanyal et al. in 2004 [20]. The subsequent trial, which was more robust in participants, reflected that pioglitazone was the most superior in reducing ALT levels among all other interventions, including placebo, and that the changes were significant between the groups (p<0.001) [24]. In the FLIRT trial by Ratviu et al., rosiglitazone was discovered to exhibit normalization of ALT levels when compared to placebo (38% vs. 7%; P= 0.005) [33]. Belfort et al. demonstrated that diet and pioglitazone were superior to diet and placebo when compared, particularly in the normalization of transaminase level (40% vs. 21%; P=0.04). This observed reduction in plasma transaminase level was also seen in the placebo arm, but the placebo arm that experienced normalization of transaminases had persistence of NASH-associated histologic abnormalities [21]. This finding is also supported by Sharma et al.; consequently, this gives merit that advanced histologic changes in NASH can occur despite normal enzyme levels or improvement in transaminase levels. Improvement in enzyme levels does not parallel amelioration of NASH/NAFLD.

Insulin Sensitivity

The homeostatic model assessment-insulin resistance (HOMA-IR) was used by most trials in projecting the effect thiazolidinediones had on patients with NASH. Insulin resistance and adipocytokines have been implicated as main players in the pathophysiology of fat tissue accumulation in the liver and attendant pathological entities in the spectrum of NAFLD. Thiazolidinediones act as activators of peroxisome proliferator-activated receptor-γ (PPAR-γ); a nuclear receptor that is predominantly expressed in adipose tissue. It is also expressed in the liver and muscle tissue. In the study by Ratziu et al., there was a reduction in insulin resistance, while an increase was observed in the placebo group [23]. Insulin resistance was also reduced in the studies by Sanyal et al. and Anushiravani et al. [24,28]. In the study by Aithal et al., there was an ostensible increase in HOMA-IR in the pioglitazone group, but this finding was counter-intuitive and was unsurprisingly not statistically significant [22]. The researcher postulated that this strange finding may be due to the participants being non-diabetics and a low dose of pioglitazone being used. However, the C-peptide levels in the pioglitazone arm were found reduced, suggesting the fact that the drug works in a way that increases insulin sensitivity as we know. In their study, Promrat et al. established these findings as both serum insulin and C-peptide levels were reported to be reduced [25]. Adiponectin levels correlate positively with insulin sensitivity. This will account for the statistically significant increase in adiponectin post-pioglitazone therapy seen by Aithal et al. in their study [22]. The very robust trial by Belfort et al. also reported the following statistically significant reduction in insulin concentration by 34% after pioglitazone therapy. In addition, serum-free fatty acid levels were also decreased, signaling a marked increment in insulin sensitivity [21].

Adverse Effects

Weight gain is an established side effect of thiazolidinediones due to their mechanism of action. It is therefore no surprise that Belfort et al. found a modest weight gain and an increase in body fat in the pioglitazone group [21]. In all pioglitazone trials, the BMI of the control groups was observed to be increased. This was not the case in Belfort et al. [21]. The only trial that involved rosiglitazone observed a significant increase in weight between the rosiglitazone arm versus the placebo arm (+1.5 kg vs. −1.0 kg, P<0.01). In this trial, it was also noted that after at least a year of therapy with rosiglitazone, a minimum of a 3 kg increment in weight was observed in about 30% of patients [23]. The insight gotten from this study provides clarity on the benefits of thiazolidinediones among patients with NAFLD. This will hopefully positively influence the clinical guidelines for preventing and treating NAFLD/NASH.

Limitations

This study is unfortunately limited by the diversity of patient characteristics and the fact that most studies employed the use of a small number of subjects, which could be attributed to the fact that the most specific diagnosis for NAFLD/NASH is via liver biopsy. In addition, this review utilized only RCTs, which is a potential obstruction to the potential conclusions found by this study.

## Conclusions

The increasing prevalence of NAFLD/NASH warrants more attention from the medical world due to its known potential as a notorious contributor to the incidence of cirrhosis. This implies that we must pay close attention to alleviating the significant players in the progression of this disease, e.g., DM, obesity, etc. It is therefore intuitive to hypothesize that thiazolidinediones should enhance insulin sensitivity. Still, we have established that there is more than just insulin resistance involved in the emanation of NAFLD/NASH; it is a complex interplay, and this must be appreciated and incorporated as we search for therapeutics that would halt the progression of this entity to fatal sequelae like cirrhosis and HCC. Perhaps combining thiazolidinediones with other hepatotropic medications would yield much-desired results; for this reason, more effort should be concentrated in this regard. In this review, we established that thiazolidinediones do improve histological and biochemical metrics for judging hepatic function in patients with NAFLD. However, this group of pharmacotherapy is linked with several adverse effects like weight gain, exacerbation of heart failure, osteoporosis, and possible attendant fractures, which may actually be a result of co-morbid cofounders, i.e., diabetes. Therefore, in order to balance the efficacy of this drug class against the concerning known side effects, more extensive clinical trials will prove beneficial as they may reveal new realities since this drug has shown significant promise thus far.
